# Prevalence Estimate of Blood Doping in Elite Track and Field Athletes During Two Major International Events

**DOI:** 10.3389/fphys.2020.00160

**Published:** 2020-02-25

**Authors:** Raphael Faiss, Jonas Saugy, Alix Zollinger, Neil Robinson, Frederic Schuetz, Martial Saugy, Pierre-Yves Garnier

**Affiliations:** ^1^Research and Expertise in Anti-Doping Sciences, Institute of Sport Sciences, University of Lausanne, Lausanne, Switzerland; ^2^Bioinformatics Core Facility, SIB Swiss Institute of Bioinformatics, Lausanne, Switzerland; ^3^Swiss Laboratory for Doping Analyses, University Centre of Legal Medicine, Lausanne and Geneva, Centre Hospitalier Universitaire Vaudois and University of Lausanne, Lausanne, Switzerland; ^4^Center for Integrative Genomics, University of Lausanne, Lausanne, Switzerland; ^5^Athletics Integrity Unit, Antidoping and Medical Department, Monaco, Monaco

**Keywords:** blood, doping, prevalence, hematological passport, athletes

## Abstract

In elite sport, the Athlete Biological Passport (ABP) was invented to tackle cheaters by monitoring closely changes in biological parameters, flagging atypical variations. The hematological module of the ABP was indeed adopted in 2011 by World Athletics (WA). This study estimates the prevalence of blood doping based on hematological parameters in a large cohort of track and field athletes measured at two international major events (2011 and 2013 WA World Championships) with a hypothesized decrease in prevalence due to the ABP introduction. A total of 3683 blood samples were collected and analyzed from all participating athletes originating from 209 countries. The estimate of doping prevalence was obtained by using a Bayesian network with seven variables, as well as “blood doping” as a variable mimicking doping with low-doses of recombinant human erythropoietin (rhEPO), to generate reference cumulative distribution functions (CDFs) for the Abnormal Blood Profile Score (ABPS) from the ABP. Our results from robust hematological parameters indicate an estimation of an overall blood doping prevalence of 18% in 2011 and 15% in 2013 (non-significant difference) in average in endurance athletes [95% Confidence Interval (CI) 14–22 and 12–19% for 2011 and 2013, respectively]. A higher prevalence was observed in female athletes (22%, CI 16–28%) than in male athletes (15%, CI 9–20%) in 2011. In conclusion, this study presents the first comparison of blood doping prevalence in elite athletes based on biological measurements from major international events that may help scientists and experts to use the ABP in a more efficient and deterrent way.

**What are the new findings?**

• This study presents the first comparison of blood doping prevalence in elite track and field athletes based on biological measurements from major international events.
• Our results from robust hematological parameters indicate an estimation of an overall blood doping prevalence of 15–18% in average in endurance athletes.
• The confidence intervals for blood doping prevalence range from 9 to 28% with wide discrepancies between certain countries.

**How might it impact on clinical practice in the near future**

• The further development of the Athlete Biological Passport with a careful monitoring of biological parameters still represents the most consistent approach to thwart athletes using undetectable prohibited substances or methods.
• This study describes a method to define blood doping prevalence with the analysis of robust hematological parameters.
• Estimates of doping prevalence in subpopulations of athletes may represent a valuable tool for the antidoping authorities to perform a risk assessment in their sport.

## Introduction

The true prevalence of doping among athletes competing at the highest level remains virtually unknown while few attempts to address this point exist (Scarpino et al., 1990; Thevis et al., 2008; Striegel et al., 2010; Sottas et al., 2011a; Ulrich et al., 2018). Prevalence of doping in sports is influenced by many cultural, environmental (e.g., climate, altitude, etc.) or social factors, and the efficiency of the anti-doping strategy is an important feature influencing this prevalence (Sjoqvist et al., 2008). In fact, official adverse or atypical results occur in less than 2% of the tests performed in laboratories accredited by the World Anti-Doping Agency (WADA) (WADA, 2018). However, such statistic is flawed and does not allow an estimate of the prevalence of doping in athletes for at least two reasons. First, because drug tests give priority to specificity rather than sensitivity, false-negative results lead to underestimate the true values because of a lack of sensitivity (Sottas et al., 2011a). In addition, cheats using low dosages of doping substances result in very short detection windows (de Hon et al., 2015). Second, some tests (e.g., in competition or at random) may have a primary deterrent effect rather than being able to detect cheats immediately. Surveys of athletes may represent an attractive alternative while truthful answers from top-level athletes tempted to deflect any suspicions toward themselves or their sport are far from guaranteed. For example, doping (in all forms) prevalence is said to range between 39 and 62% based on anonymous questionnaires answered by athletes competing in two 2011 competitions of the World Athletics [WA, formerly International Association of Athletics Federations (IAAF)] (Ulrich et al., 2018). In the latter study, the randomized response technique was hence utilized since it is proposed to improve the quality of data gathered especially for sensitive questions like doping (de Hon et al., 2015; Pielke, 2018). Notwithstanding the surprisingly big values, these results shall first underline the large variability and heterogeneity in the determination of doping prevalence with a questionable significance.

In another way, performance data from athletes convicted for doping violations was used to assess the predictive performance of a Bayesian framework with a probit model. Briefly, Bayesian inference is a method by which the probabilities of various hypothetical causes are computed from the observation of known events. For example, such a model was able to detect performance differences between doped and presumed clean shot put athletes (Montagna and Hopker, 2018). The latter supports the robustness of objective data (e.g., measurable performance or hematological variables) for an unbiased estimate of doping prevalence in sport.

In this context, monitoring an athletes’ hematological parameters is a smart concept allowing to track individual changes over time with discrepancies naturally due in a certain range to physiological changes and potentially due to any external cause (medical condition or doping) over a certain limit. Such a concept of longitudinal monitoring of blood parameters was conceived in parallel to direct detection methods with a mathematical model to identify biological markers indicative of doping with the Athlete Biological Passport (ABP) (Striegel et al., 2010; Saugy et al., 2014; Salamin et al., 2017). The ABP is thus based on a longitudinal approach of individual changes in selected biomarkers using a Bayesian statistical method. Nowadays, the high standardization of the blood tests according to ABP guidelines published by the WADA (2009) would allow a reliable estimation of blood doping prevalence through epidemiological measures of occurrence. For example, the Abnormal Blood Profile Score (ABPS) is calculated and quantified in the ABP. The ABPS is calculated based on a combination of reticulocyte percentage (RET%), red blood cell count (RBC), hemoglobin (HGB), hematocrit (HCT), mean corpuscular volume (MCV), mean corpuscular hemoglobin (MCH), and mean corpuscular hemoglobin concentration (MCHC) (Sottas et al., 2007; Schütz and Zollinger, 2018). This score has been successfully used to estimate the prevalence of blood doping in elite track and field athletes (Sottas et al., 2011a).

Indeed, the WA targeted top-level track and field athletes with complete blood testing programs as early as in 2001 (resulting in the latter study) and adopted the ABP in 2011 after it was first introduced in cycling in 2009. For instance, blood tests for all athletes participating in the 2011 WA World Championships in Daegu (South Korea) served to build a solid reference basis for hematological, steroidal, and endocrines modules in these athletes (Robinson et al., 2012). The WA decided to announce this exceptional testing program prior to the event and repeated the program in the 2013 World Championships in Moscow (Russia) with all athletes participating being tested for blood parameters. A thorough description of these data allowed the recent publication of a worldwide distribution of blood values in elite track and field athletes (Robinson et al., 2019). All blood parameters determined from these samples collected during the events were then introduced in each athlete’s individual hematological module of the ABP for further analysis.

Consequently, this study aims to analyze the data collected during the 2011 and 2013 events and to present estimates of the prevalence of doping in participating athletes based on the evaluation of specific blood variables determined for the ABP. With the individual longitudinal monitoring of biological variations implemented in 2011 by WA (i.e., the ABP to scrutinize variations in blood parameters), it is hypothesized to observe a decrease in this doping prevalence between 2011 and 2013. Additionally, the study allows an estimate of the prevalence of blood manipulations, not only in the entire population of endurance athletes, but also in sub-groups (countries) participating to these two competitions. This approach provides important information to the antidoping authority to elaborate an appropriate antidoping policy.

## Materials and Methods

### Sample Collection and Biological Analyses

A total of 3683 blood samples were collected and analyzed during WA World Championships: 1808 in Daegu (South Korea, 2011) and 1875 in Moscow (Russia, 2013). All athletes originating from 209 countries were tested before the competition. In case some athletes were tested more than once during the competition period, only the first record was kept for our analysis. Blood sampling took place between 07:05 and 24:00 o’clock. A detailed description of the athletes included and the resulting samples included in this study are described in detail elsewhere (Robinson et al., 2019). Due to the design of the study collecting blood samples in all athletes who competed in two major events, the dataset represents the most comprehensive population possible (maximal sample size). The lack of sample size analysis is justified by the anti-doping perspective of our work hypothesis aimed at avoiding false-positives (high specificity), even though this may result in false-negatives (lower sensitivity). As such, we acknowledge that we may be unable to identify some countries with a non-zero prevalence of doping because sufficient power to identify the effect is lacking due to the sample size limited by the design of the study itself. Sample collection procedures, preconditioning, analysis, and storage have been thoroughly described for the 2011 event in Daegu (Robinson et al., 2012) and precisely reproduced in 2013 in Moscow in order to allow for a comparative analysis of the results. However, for convenience to the reader, some key points to set the context of the present study have to be introduced. For the events, a mobile WADA-accredited laboratory unit was created including several blood-collection stations. Athletes were requested to report to a designated station within 24 h upon their arrival on the competition sites as part as the routine anti-doping procedure. All blood tests were done following the WADA ABP operating guidelines (WADA, 2009) and tubes were stored immediately in monitored fridges before transportation to the on-site analytical laboratory in an insulated cool box with a controlled temperature of approximately 4°C (as recorded by a temperature datalogger). All samples were analyzed within less than 24 h after blood withdrawal with one of the two identical hematological analyzers (Sysmex XT-2000i, Sysmex Europe, Norderstedt, Germany) required to manage the high number of samples. In Daegu, the mobile unit was managed by a team from the Lausanne WADA accredited laboratory whereas in Russia, the analyses were conducted by the Moscow WADA accredited laboratory, in accordance with the related WADA technical document (WADA, 2009). All samples and blood tests were conducted in accordance with the ethics code of conduct of WADA and the study was approved by WADA’s Ethical Committee as regulator of any sample collected in an antidoping context. Subjects signed an informed consent that the collected sample and the doping control related data would be used for anti-doping research purposes provided that they could no longer be identified.

**FIGURE 1:**
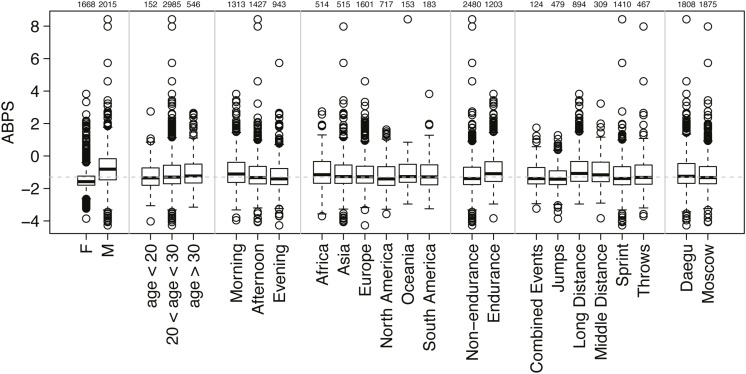
Distribution of all individual ABPS values obtained with the distinction of seven sub-classifications (sex, age, sampling time, origin of athlete, endurance or non-endurance disciplines, disciplines, and competitions). The number of athletes in each subgroup is indicated above the graph. The boxes of the boxplots represent scores between 25 and 75%, with the median indicated with a bold line in the box; outliers are scores >1.5 times the interquartile range (75–25%, the length of the box) from the box and are indicated by dots; whiskers extend to the highest or lowest not considered to be an outlier.

The ABPS was calculated from seven hematological variables: RET%, HGB, HCT, RBC, MCV, MCH, and MCHC (Sottas et al., 2006; Schütz and Zollinger, 2018). The dataset used for the prevalence estimate in the present study is illustrated by the distribution of all individual ABPS values obtained in Daegu and Moscow with the distinction of the subsequent classifications described below and illustrated in Figure 1 (as published in Robinson et al., 2019).

### Estimation of Prevalence and Statistical Analysis

The ABPS of the population studied was compared with a simulated reference athletic population, based on the population described in Robinson et al. (2019). The reference population is characterized by seven heterogeneous variables, namely endurance, age, sex, ethnicity (i.e., continent), altitude, disease (i.e., if athletes reported being sick), and instrument used for the analysis.

Athletes were first classified into “endurance” and “non-endurance”; “endurance” comprised all athletes competing in running or walking events with distances equal or longer than 800 m while “non-endurance” included all athletes competing in jumps, sprints, throws, and combined events, as well as distances shorter than 800 m.

Second, age at sampling collection allowed a classification in the three following categories for all athletes: ≤19 years, 19–24 years, and ≥25 years, while males and females were separated as such.

Then ethnicity was defined with four categories (Caucasian, Asian, African, and Oceanian). Since only information about the country of origin of the athletes was available, proportions were estimated for countries having more than 10 athletes based on the Central Intelligence Agency World Factbook (CIA, 2018). While such statistics concern the general population, it still provides a fair estimate of athletes representing those countries and should not be considered as a bias to the results. Countries with less than 10 athletes were grouped by continent with imposed proportions of ethnicity (ordered as Caucasian, Asian, African, and Oceanian) per continent as follows: Europe = (1, 0, 0, and 0), Asia = (0, 1, 0, and 0), Africa = (0, 0, 1, and 0), North America = (0.48, 0.07, and 0.45, 0), South America = (0.25, 0.25, 0.25, and 0.25), and Oceania = (0, 0, 0, and 1).

Altitude exposure before the events was considered and differentiated between endurance and non-endurance athletes with an allocation of athletes between categories: <1000, 1000–1500, 1500–2000, and >2000 m. Because no information about prior altitude exposure was available in Daegu, following proportions in the respective categories described above were arbitrarily applied to endurance athletes (0.5, 0.2, 0.2, and 0.1) and non-endurance athletes (0.96, 0.02, 0.01, and 0.01). Since prior altitude exposure data was only partially recorded in Moscow, athletes were allocated to the same categories with the following proportions: endurance (0.5329, 0.1557, 0.1557, and 0.1557) and non-endurance (0.96, 0.0133, 0.0133, and 0.0133). All athletes were assumed to be healthy (i.e., not sick at the moment of sample collection) and non-smoking.

**FIGURE 2:**
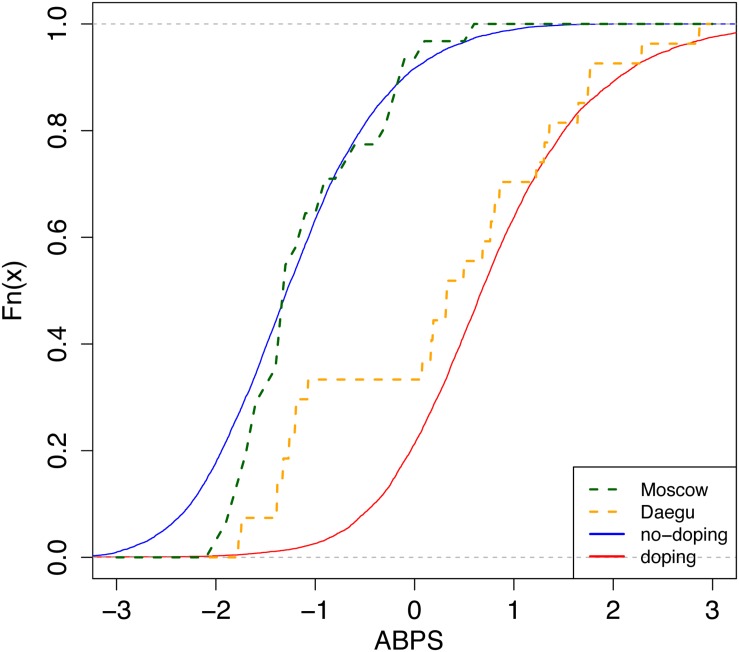
Cumulative distribution functions (CDFs) of the Abnormal Blood Profile Score (ABPS) marker as calculated in the Athlete Biological Passport indicating doping prevalence for endurance athletes from country N. Solid lines: reference CDFs obtained for a modal population of endurance athletes; blue: assuming no-doping, red: assuming doping with microdoses of rhEPO (Ma et al., 2008; Sottas et al., 2008, 2011a). The difference between both lines refers to the discriminative power of the ABPS marker. Dashed lines: empirical CDFs obtained from all tests performed in endurance athletes in Daegu (orange, *n* = 27) and Moscow (green, *n* = 31).

**FIGURE 3:**
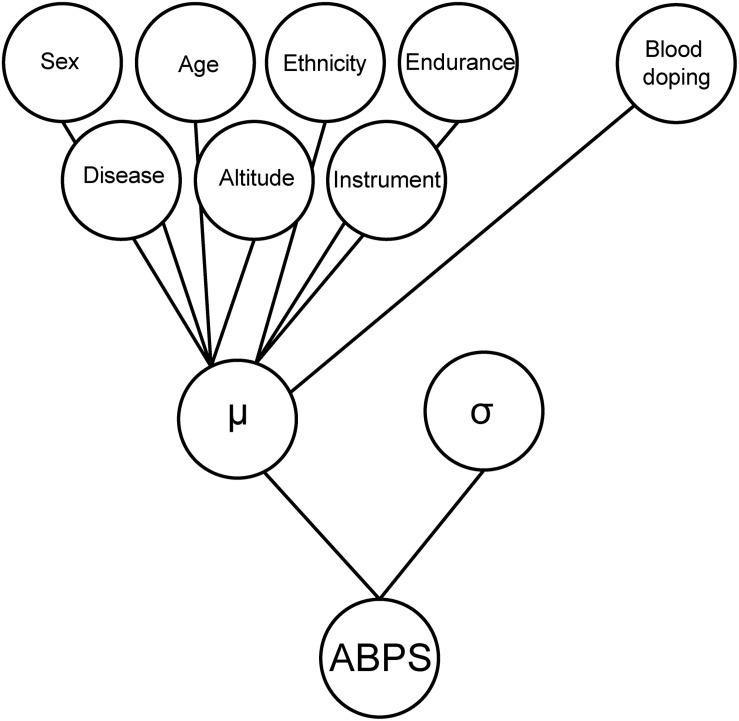
Graphical illustration of the Bayesian model used for the marker “ABPS” including the seven heterogeneous factors (hard evidence) as well as “blood doping” as a variable mimicking doping with low-doses of rhEPO. The heterogeneous factors are entered with a linear effect on the individual mean (μ) and the model adjusts for the standard deviation (σ) of the ABPS marker.

To estimate the prevalence of doping in different populations, a Bayesian network was used with the seven variables described above, as well as “blood doping” as a variable mimicking doping with low-doses of recombinant human erythropoietin (rhEPO), to generate the simulated reference population, which is then used to generate reference cumulative distribution functions (CDFs, solid curves from Figure 2 for the marker ABPS). The Bayesian model used for the marker “ABPS” (Figure 3) illustrates the seven heterogeneous factors (hard evidence) as well as “blood doping” [see Supplementary Material in Sottas et al. (2011a)]. When the ABPS is known in an individual athlete (from the seven hematological variables measured), the factor “blood doping” is estimated using Bayesian inference to produce two reference curves of “doping” and “no-doping” including the heterogeneous factors entered. In a Bayesian perspective, the reference curves reflect the prior predictive ABPS value that indicate what the data should look like (in case of doping or no-doping) and a known ABPS value may in turn allow to calculate a probability of doping with the heterogeneous factors entered.

The generation itself of CDFs is indeed well described elsewhere (Sottas et al., 2011a). Briefly, the prevalence is estimated from the CDF as the ratio of two areas: (1) the area between the reference curve of no-doping (solid and left) and the ABPS curve (dashed), and (2) the area between the two reference curves (solid, left, and right) (see Figure 2).

Besides, in the original paper (Sottas et al., 2011a), no difference in the variation of the ABPS between men and women was assumed. However, in our study, we observed a striking difference. We therefore introduced a correction factor for sex in the Bayesian network. In addition, reference values were adjusted for the mean and standard deviation (μ, σ of the doping and non-doping reference curves of ABPS, Figure 2) in order to correspond to the observed ABPS score. A systematic shift was observed in all ABPS values measured in Moscow compared to the values measured in Daegu (−0.16); since such a shift is not related to a particular biological reason, it was corrected using the adjustment factor determined from the reference values described in Robinson et al. (2019). All factors included to adjust ABPS values (sex, age, time of sampling, athlete origin, and competition) are reported in Table 1. Since the seven hematological variables are related, only HGB, HCT, RBC, and RET% were corrected for this pre-analytical bias, using the following additive factors: HGB −0.3, HCT −0.7, RBC −0.08, and RET% +0.07. The three other variables were calculated as MCV = 10 × HCT/RBC, MCH = 10 × HGB/RBC, and MCHC = 100 × HGB/HCT.

**TABLE 1:** Adjustment coefficients according to a simple linear model for the ABPS.

	Factor	95% CI
Female	−0.71	−0.76 to −0.65
Age < 20	−0.01	−0.15–0.14
Age > 30	0.05	−0.03–0.13
Afternoon	−0.18	−0.25 to −0.11
Evening	−0.28	−0.35 to −0.21
Africa	−0.12	−0.21 to −0.03
Asia	−0.04	−0.13–0.05
North America	−0.17	−0.25 to −0.09
Oceania	−0.08	−0.22–0.07
South America	−0.04	−0.17–0.09
Moscow	−0.16	−0.22 to −0.1

*CI, confidence interval.*

Finally, the time of the day the blood samples were collected induced non-analytical variations in the ABPS. Values of the ABPS for all athletes whose blood samples were taken in the afternoon or in the evening (reference is set to be morning sampling) were additionally corrected. The reference curve corresponds to non-endurance athletes; to ensure that the corrections described above were required and not linked to measurement bias, linear models were used to make sure that the correction factors were the same for endurance and non-endurance athletes (data not shown).

The estimated doping prevalences at Daegu and Moscow were compared using a Kolmogorov–Smirnov test, which assesses the largest vertical difference between the two curves, and a Cramér–von Mises test, which considers the sum of the differences. The *p*-values obtained were adjusted for multiple testing using the Benjamini–Hochberg procedure to control the false discovery rate (Benjamini and Hochberg, 1995). The 95% confidence intervals (CIs) for prevalence were obtained by resampling and constructed based on 1000 bootstrap estimates from the observed data. While the latter analysis is inappropriate in case of zero estimates and may lack sufficient power in the case of small sample sizes, the inter-athlete variability within our population did not produce exact zero estimates and the applied resampling method may thus still provide pertinent results in the particular scenario of our study.

It is worth noting that the use of simulated reference populations (and the absence of an ideal reference population with a known zero prevalence of doping), combined with the inter-variability of measurements across different athletes, can cause some estimates of variability (as well as the extremities of some of the CIs) to be negative. We acknowledge that negative prevalence estimates are impossible to achieve. However, if a population has a 0% estimate of doping prevalence, ABPS values from the subset population below the median value would produce a negative estimate. The other half of the population would in turn have a positive estimate of prevalence, thus explaining the null prevalence for the entire population.

All calculations and analyses were performed using the R software (R Development Core Team, 2005). Values for the ABPS were calculated using a custom-made R package described elsewhere (Schütz and Zollinger, 2018). Please see Supplementary Material for a step-by-step methods description.

## Results

### Description of the Population Analyzed

Table 2 presents the distribution of samples across sex, age, continent, sport, and altitude variables for WA World Championships in Daegu (2011) and Moscow (2013). Each variable is divided in subcategories. NCC America stands for North America, Central America and Caribbean. A total of 3683 samples were tested, among those the proportions of males versus females were almost equal. In addition, approximately one third were competing in endurance disciplines.

**TABLE 2:** Proportions of samples for each variable for Daegu (2011) and Moscow (2013) WA World Championships.

Variable	Categories	Daegu (*n* = 1808)	Moscow (*n* = 1875)
Sex	Male	53%	56%
	Female	47%	44%
Age	≤19	2%	2%
	19–24	38%	40%
	≥25	60%	58%
Continent	Africa	15%	14%
	Asia	16%	12%
	Europe	42%	44%
	NCC America	19%	20%
	Oceania	4%	4%
	South America	4%	5%
Sport	Endurance	31%	35%
	Non-endurance	69%	65%
Altitude	<1000 m	NA	81%
	>1000 m	NA	19%

*NCC America stands for North America, Central American, and Caribbean. NA, no adequate data available.*

### Prevalence of Doping: Endurance Only

Table 3 presents the prevalence of blood doping along with 95% CIs in all endurance athletes for Daegu and Moscow (569 athletes in Daegu and 653 athletes in Moscow), stratified by sex or country. Only the 18 countries with at least 10 athletes competing in endurance sports at either Daegu or Moscow competitions are represented. The overall prevalence of blood doping does not decrease significantly between Daegu and Moscow (0.18 to 0.15, NS). The overall prevalence of doping between the two competitions indicates a non-significant decrease in female athletes (0.22 to 0.12, NS) while a non-significant increase is observed in male athletes (0.15 to 0.17, NS). Among the selected countries, eight tend to decrease their prevalence of blood doping between 2011 and 2013 WCS (only significantly for countries N and Q, *P* < 0.001), eight increase (significantly only for country L) and two of them stay at the same level (0.00 for countries H and I in both competitions).

**TABLE 3:** Prevalence of blood doping along with 95% confidence intervals.

	2011 Daegu			2013 Moscow				
		
Sex and country	*n*	Prevalence	CI	*n*	Prevalence	CI	p.adj ks	p.adj cvm
All	569	0.18	0.14–0.22	653	0.15	0.12–0.19	0.80	0.31
Female	246	0.22	0.16–0.28	276	0.12	0.09–0.16	0.80	0.31
Male	323	0.15	0.09–0.2	377	0.17	0.12–0.22	0.80	0.31
Country A	15	0.00	−0.22–0.09	22	0.04	−0.12–0.2	0.98	0.50
Country B	11	0.15	−0.16–0.45	8	0.02	−0.24–0.27	0.98	0.48
Country C	6	0.32	0.02–0.61	18	0.07	−0.11–0.25	0.93	0.31
Country D	8	0.17	−0.04–0.39	10	0.31	0.06–0.56	0.99	0.63
Country E	33	0.19	0.09–0.3	37	0.30	0.17–0.43	0.98	0.50
Country F	10	0.03	−0.2–0.28	14	0.17	0–0.34	0.98	0.53
Country G	16	0.04	−0.1–0.2	14	0.18	−0.04–0.42	0.98	0.30
Country H	7	0.00	−0.47–0.02	18	0.00	−0.14–0.14	0.99	0.63
Country I	25	0.00	−0.22–0	20	0.00	−0.27–0.2	0.98	0.31
Country J	43	0.19	0.06–0.32	42	0.13	0–0.26	0.99	0.63
Country K	17	0.47	0.17–0.8	19	0.61	0.34–0.86	0.98	0.53
Country L	16	0.00	−0.22–0.09	18	0.27	0.07–0.48	0.80	0.04
Country M	27	0.15	0.01–0.28	23	0.09	−0.06–0.23	0.99	0.61
Country N	27	0.74	0.48–0.99	31	0.09	−0.03–0.2	<0.001	<0.001
Country O	10	0.66	0.18–1.14	6	0.23	−0.12–0.58	0.98	0.31
Country P	10	0.17	−0.13–0.45	11	0.11	−0.11–0.31	0.98	0.53
Country Q	19	0.89	0.63–1.14	16	0.25	0.03–0.48	0.80	0.01
Country R	41	0.14	0.03–0.25	41	0.17	0.06–0.28	0.99	0.57

*The prevalences for the countries are given for endurance athletes only. The “*n*” columns give the number of athletes for each category for Daegu and Moscow competitions. Only countries with more than 10 athletes competing in endurance sports at either Moscow or Daegu competitions are presented. CI, confidence interval; p.adj ks, *p*-value for the adjusted Kolmogorov–Smirnov test; p.adj cvm, *p*-value for the Cramér–von Mises criterion test.*

### Prevalence of Doping: Endurance Only for Female Athletes

Table 4 presents the prevalence of blood doping along with 95% CIs in female athletes for Daegu and Moscow. For pertinence and anonymity, only countries with at least seven athletes or more competing in endurance sports at either Daegu or Moscow competitions are represented. As said before, the overall prevalence of doping for endurance women only tend to decrease between the two competitions (0.22 to 0.12 for Daegu and Moscow respectively, NS). Among selected countries, only four show a decrease in the prevalence of blood doping between 2011 and 2013 [only significant for countries N (*P* < 0.001), and Q (*P* < 0.01)], five tend to increase (not significantly), one stays at the same level (country I), and three of them do not have sufficient participants to make a prevalence calculation in one of the competitions.

**TABLE 4:** Prevalence of blood doping along with 95% CIs for endurance female athletes only.

	2011 Daegu			2013 Moscow				
		
	*n*	Prevalence	CI	*n*	Prevalence	CI	p.adj ks	p.adj cvm
Female endurance	246	0.22	0.16–0.28	276	0.12	0.09–0.16	0.83	0.27
Country A	4			11	0.00	−0.11–0.05		
Country C	2			7	0.05	−0.11–0.19		
Country E	16	0.21	0.1–0.33	19	0.22	0.15–0.3	0.93	0.70
Country G	10	0.00	−0.04–0.04	8	0.08	0.02–0.14	0.83	0.22
Country I	13	0.00	−0.1–0.05	7	0.00	−0.19–0.12	0.93	0.54
Country J	21	0.10	−0.04–0.24	19	0.09	−0.05–0.23	0.93	0.70
Country K	6	0.41	−0.23–1.03	7	0.24	−0.08–0.56	0.93	0.70
Country L	9	0.00	−0.15–0.06	10	0.19	0.01–0.39	0.83	0.22
Country M	8	0.17	0.02–0.33	7	0.30	0.11–0.47	0.83	0.22
Country N	15	0.73	0.43–1.02	20	0.08	−0.04–0.2	0.03	<0.001
Country O	8	0.63	0.12–1.14	3				
Country Q	16	0.91	0.62–1.21	7	0.31	−0.06–0.66	0.21	<0.001
Country R	21	0.13	0.01–0.24	22	0.19	0.11–0.27	0.83	0.22

*The “*n*” columns give the number of athletes for each category for Daegu and Moscow competitions. Only countries with more than seven athletes competing in endurance sports at either Moscow or Daegu competitions are presented. CI, confidence interval; p.adjks, *p*-value for the adjusted Kolmogorov–Smirnov test; p.adj cvm, *p*-value for the Cramér–von Mises criterion test.*

### Prevalence of Doping: Endurance Only for Male Athletes

Table 5 presents the prevalence of blood doping along with 95% CIs in male athletes for Daegu and Moscow. Again, for pertinence and anonymity, only countries with at least 10 athletes or more competing in endurance sports at either Daegu or Moscow competitions are represented. The overall prevalence of blood doping for men in endurance tends to rise between Daegu and Moscow (0.15 versus 0.17, NS). Among selected countries four tend to decrease their prevalence of blood doping between 2011 and 2013 (only significantly for country N), six tend to increase (only significantly for country L), one stays at the same level (country I, prevalence 0.00), and two of them do not have sufficient participants to make a prevalence calculation in one of the competitions.

**TABLE 5:** Prevalence of blood doping along with 95% CIs for endurance male athletes only.

	2011 Daegu			2013 Moscow				
		
	*n*	Prevalence	CI	*n*	Prevalence	CI	p.adj ks	p.adj cvm
Male endurance	323	0.15	0.09–0.2	377	0.17	0.12–0.22	0.84	0.39
Country A	11	0.00	−0.24–0.12	11	0.12	−0.11–0.36	0.84	0.22
Country C	4			11	0.08	−0.15–0.31		
Country D	8	0.17	−0.04–0.39	9	0.34	0.11–0.57	0.84	0.39
Country E	17	0.17	0.05–0.29	18	0.39	0.22–0.55	0.84	0.20
Country G	6	0.12	−0.12–0.37	6	0.33	−0.02–0.67	0.84	0.27
Country I	12	0.00	−0.38 to −0.01	13	0.00	−0.35–0.28	0.84	0.39
Country J	22	0.27	0.09–0.43	23	0.17	0.01–0.32	0.84	0.22
Country K	11	0.51	0.21–0.81	12	0.83	0.62–1.06	0.84	0.23
Country L	7	0.00	−0.33–0.16	8	0.39	0.21–0.56	0.34	0.01
Country M	19	0.14	−0.03–0.3	16	0.00	−0.19–0.19	0.84	0.39
Country N	12	0.75	0.37–1.14	11	0.10	−0.08–0.28	0.08	<0.001
Country Q	3			9	0.21	−0.08–0.5		
Country R	20	0.16	0.03–0.29	19	0.15	−0.01–0.32	0.92	0.57

*The “*n*” columns give the number of athletes for each category for Daegu and Moscow competitions. Only countries with more than six athletes competing in endurance sports at either Moscow or Daegu competitions are presented. CI, confidence interval; p.adj ks, *p*-value for the adjusted Kolmogorov–Smirnov test; p.adj cvm, *p*-value for the Cramér–von Mises criterion test.*

### Comparison of Prevalence Between Daegu and Moscow

**FIGURE 4:**
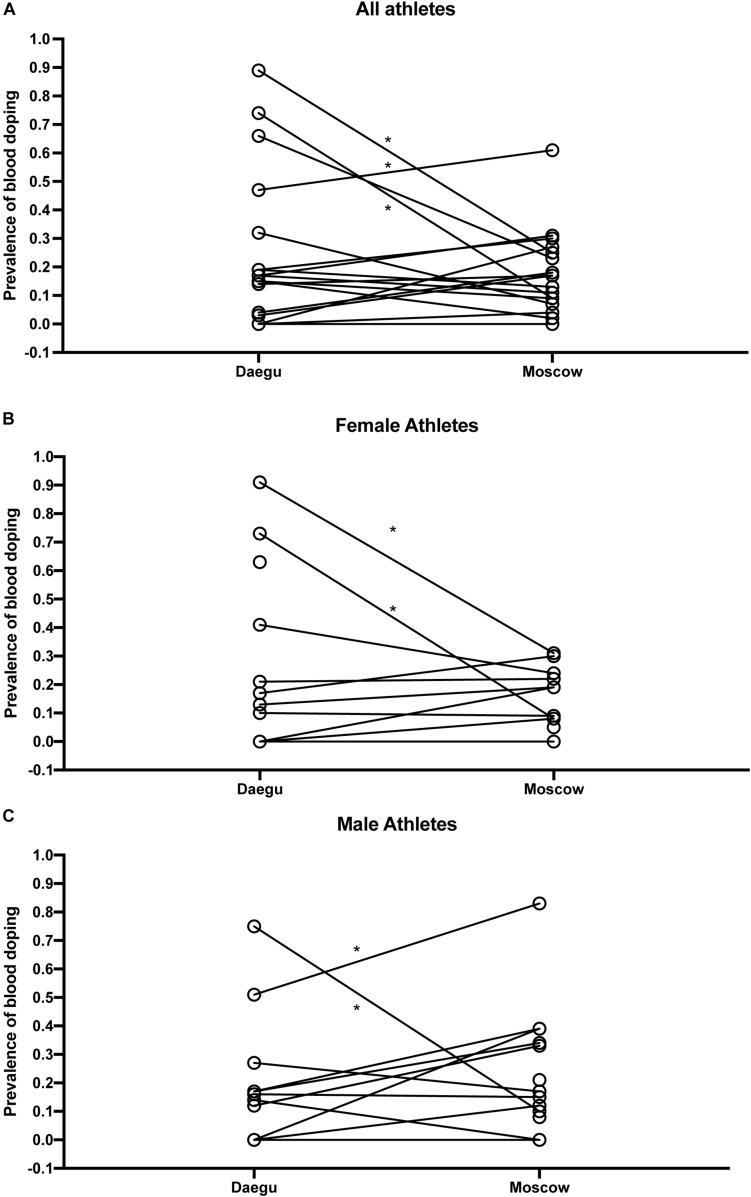
Comparison of blood doping prevalence between Daegu (2011) and Moscow (2013) from selected countries without any sex difference **(A)**, in endurance female athletes only **(B)**, and in endurance male athletes only **(C)**. *P < 0.05 for the difference with Daegu.

Overall, Figure 4 presents the comparison of blood doping prevalence between Daegu (2011) and Moscow (2013). Figure 4A illustrates prevalence from selected countries without any sex difference, Figure 4B in endurance female athletes only, and Figure 4C in endurance male athletes only.

## Discussion

This study presents for the first time a comparison of the prevalence of doping based on blood samples analyzed in all athletes competing in two top-level athletic held in 2011 and 2013. The overall estimate of blood doping prevalence in all athletes measured in the 2011 WA World Championships indicates that a clear majority of the athletes do not resort to blood doping (i.e., with 18% overall prevalence of doping). This result calculated from accurately determined biological parameters is contrasting with the 44% prevalence for doping in general (i.e., not specifically blood doping) obtained from the same event with a survey-based estimation (Ulrich et al., 2018). In fact, such discrepancy may be first partly explained by the fact that our estimation refers mostly to the prevalence of blood doping that may not be used by non-endurance athletes. Then, the definition of doping differs between both studies. Here we focus specifically on blood doping while Ulrich et al. (2018) assess doping in general. Indeed, one may understand the clear difference between prevalence calculated from robust biological parameters or self-reported surveys. On one hand, biological variations of hematologic parameters were observed from very strict, controlled and repeatable measurements (thus eliminating acquisition biases). On the other hand, confusion between medication misuse and real doping behavior for athletes originating from 200 different countries might not be excluded with the analysis of surveys. Blood doping is defined by the WADA as the misuse of certain techniques and/or substances to increase one’s red blood cell mass. An augmented hemoglobin mass is indeed associated with enhanced aerobic performance (Hauser et al., 2016; Saugy et al., 2016) with improved oxygen transport increasing endurance and stamina. The use of an injected erythropoietin stimulating agent (ESA) and blood transfusion (Salamin et al., 2016) may represent the most widespread strategies of blood doping (Elliott, 2008; Macdougall and Ashenden, 2009). While methods exist to detect ESAs both in blood and urine, autologous blood transfusion remains a challenge in the fight against doping (Morkeberg, 2012). While the discovery of erythropoietin (EPO) suddenly made blood doping simpler, many cheaters returned to blood transfusions upon significant development of detection methods (Schumacher et al., 2012; Salamin et al., 2017). Definitely, autologous blood transfusion is the method of choice with no valid method to date to accurately directly detect such intervention (Malm et al., 2016). Monitoring variations in blood parameters in athletes as implemented with the ABP may, however, be observed and would allow to flag abnormal alterations. It was therefore hypothesized that the introduction of the ABP by the WA in 2011 would result in a decrease of doping prevalence evaluated at the 2013 World Championships in the third season after its introduction. Our results indicate that only two countries significantly decreased doping prevalence between 2011 and 2013, one country has a significant increase of prevalence, while the results for the other countries do not support significant changes in the blood doping prevalence. Since all athletes and tests were conducted during the same competitions and under the same protocol, any confounding factors related to procedures or analysis may be excluded. Considering Figure 2, country N produced an empirical CDF (ECDF) very close to the reference CDF representing the “doping” curve in Daegu while its ECDF during Moscow is then very similar to the reference CDF representing the no-doping case. Thus, only external effects may explain the difference between these two ECDFs because the collection, storage, and testing protocols were scrupulously the same within the two competitions.

Moreover, since athletes use doping to improve athletic performance and would in turn benefit from a competitive advantage, countries with higher prevalence may obtain better results (e.g., number of medals). For instance, one country improved its ranking among the best countries in the overall medals table despite a significant decrease in doping prevalence observed. Conversely, another country with a low prevalence in Daegu won less medals in Moscow despite a significantly higher doping prevalence. The latter underlines the loophole of prevalence estimates to evaluate competitive results of track and field athletes.

### Limitations and Strengths of the Study

Our analysis is limited to discrete “in-competition” time-points for the analysis and may not ideally highlight the use of the numerous doping substances or procedures available. While the investigation of doping prevalence is consequently challenging, the use of Bayesian network as used in our study may, however, yield sufficient power over time to discriminate individual hematological variations (Sottas et al., 2011a). At first sight, one may think that discrepancies between 2011 and 2013 for certain parameters (like HGB being lower in average by 0.3 g/dL in 2013) may add noise to the analysis. However, our approach included adjustment coefficients for such discrepancies and blood doping estimates were calculated accordingly with a careful consideration of these adjustment and the seven heterogeneous factors of our Bayesian model. The latter underlines the possibility of a robust comparison between both events even though certain confounding factors may still have an influence on the outcome. In addition, a limitation of this analysis is that the ABPS may not allow to identify all possible blood doping strategies and our results shall thus be interpreted with care. However, “the ABPS is for instance the only universal multiparametric blood doping marker that may discriminate doped from undoped athletes independently from the rhEPO administration period. Conversely, a “stimulation” index (like the “OFF-score”) may be less sensitive to “the period when the erythropoiesis is stimulated (ON-state)” (Sottas et al., 2011b).

Besides, huge differences between countries are observed with countries where doping prevalence is close to inexistent. The latter is contrasting with WADA statistics underlining adverse analytical findings also in those countries (WADA, 2018). This may however be due to stricter anti-doping policies with intelligent and efficient targeting and testing of athletes where the few athletes tempted to use doping are being caught. The number of athletes competing, which is different from one country to another, also influences our ability to assess the prevalence of doping for each country. For countries with a smaller number of athletes competing, the power for detecting differences in doping prevalence will be lower. Given that our dataset includes all athletes competing in two major track and field competitions, the sample size could not be extended. For instance, the anti-doping perspective of our approach aimed at avoiding false-positives (high specificity), even though this may result in false-negatives (lower sensitivity). As such, we acknowledge that the analysis may not identify some countries with a non-zero prevalence of doping because sufficient power to identify the effect is lacking due to the sample size limited by the design of the study itself. Moreover, one should note that the method used to determine prevalence estimates may produce a notable upward bias, because of an unavoidable sparse-data bias due to limited sample size for some countries (Greenland et al., 2016). The results for these countries shall be interpreted with care also considering the descriptive character of this investigation.

In addition, altitude exposure prior to the competitions as a confounding factor was included with due care in the analysis because of its putative influence on the interpretation of ABP data (Lobigs et al., 2018; Sutehall et al., 2019). While data about prior altitude exposure were missing for Daegu and only partially recorded in Moscow, the reference population was constructed with an allocation of 50% of the athletes to some form of prior altitude exposure. Definitely, including more accurate information on prior altitude exposure would help improve the determination of an estimate of blood doping prevalence with the proposed method.

On the other hand, one strength of this study relies in the estimate of prevalence of blood doping based on objective biological parameters. The latter represents a unique opportunity to assess abnormal variations toward a set reference population resulting in a prevalence estimate certainly with lesser bias than survey-based estimates. However, one shall acknowledge that the strength of the analysis relying on a large cohort is being limited by ethnical, sex, and environmental factors leading to a lesser power for calculations made comparing unique countries or smaller sub-groups of the cohort.

Besides, it is true that the data only reflects prevalence at the time of the events. However, the ABPS in a longitudinal approach allows for an indirect indication of blood doping. This means that athletes may not be tested positive for rhEPO that was used during training before the events but their elevated ABPS values may reflect blood doping use in the weeks prior to these events.

Finally, one should consider averaged values from all countries with care since some prevalence estimates may be misleading for countries where only a limited number of athletes could be included.

## Conclusion

In conclusion, for the first time, an International Federation chose to lead a massive blood testing campaign and announced it. Blood samples from all athletes were analyzed in both Daegu (2011) and Moscow (2013) WA World Championships. Our investigation indicates a moderate prevalence of blood doping in these athletes ranging from 12 to 22%. With the introduction of the ABP in 2011, a decrease in the prevalence of doping over time was hypothesized. However, our results do not support this hypothesis with a non-significant decrease in doping prevalence (overall from 18 to 15%) between 2011 and 2013. The further development of the ABP with a careful monitoring of biological parameters still represents the most consistent approach to thwart athletes using undetectable prohibited substances or methods. Such approach may definitely be useful to estimate doping prevalence in subpopulations of athletes, providing a tool for the antidoping authorities to perform a risk assessment in their sport.

## Data Availability Statement

The datasets generated for this study are available on request to the corresponding author.

## Author Contributions

MS, NR, and P-YG conceived the project and obtained the project funding. MS and NR contributed to the collection of the data. AZ and FS statistically analyzed the data. RF, JS, and MS drafted the final version of the manuscript. All authors contributed to revising the manuscript and expressed their approval of the final submitted version.

## Conflict of Interest

Upon manuscript submission, NR was employed by the International Testing Agency (ITA, Lausanne, Switzerland). MS and NR were employed by the LAD at the time of data collection. The analytical work by AZ and FS was funded by the LAD. The remaining authors declare that the research was conducted in the absence of any commercial or financial relationships that could be construed as a potential conflict of interest.
