# Supplementary material for: Prevalence Estimate of Blood Doping in Elite Track and Field Athletes During Two Major International Events
Source: Front Physiol. 2020 Feb 25;11:160. doi: 10.3389/fphys.2020.00160 (PMC7052379; doi:10.3389/fphys.2020.00160)
Supplement: Supplementary file 1 [file Data_Sheet_1.DOCX]

**Prevalence estimate of blood doping in elite track and field athletes**

**during two major international events**

Raphael Faiss, Jonas Saugy, Alix Zollinger, Neil Robinson, Frédéric Schütz, Martial Saugy, Pierre-Yves Garnier.

*Details of the Estimation of prevalence and statistical analysis*

All calculations and analyses were performed using the R software, version 3.3 (R Development Core Team 2005). All the additional packages that were used (as indicated in the text below) are available on the CRAN (Comprehensive R Archive Network) repository.

The original dataset was first cleaned, in order to remove measurements with missing values or multiple measurements during the same competition. ABPS values calculated using the “ABPS” R package (Schütz and Zollinger 2018) were used for the rest of the analysis. ABPS values calculated using this package have been shown to be equivalent to the values calculated within the ADAMS database.

The different variables were categorized as described in the main manuscript (endurance vs non-endurance, age, time of sample collection), and a reference population was defined, as per (Robinson et al. 2019).

In order to account for the systematic difference between the Daegu and Moscow measurements, the haematological parameters were corrected using the adjustment values described in the manuscript, which were inferred from the data using a linear model, as described in (Robinson, Saugy, Schutz, Faiss, Baume, Giraud and Saugy 2019) Similarly, the individual haematological parameters were corrected using values obtained from the data using the linear model, and the ABPS values were recalculated after adjustment.

The estimates of prevalence and differences in prevalence were then estimated by comparing the areas of curves for the observed populations and simulated reference populations. The Bayesian networks used for simulating the populations were created using the “bnlearn” package (“Bayesian network structure learning, parameter learning and inference”), as described in the main text.

Once estimated results were obtained, confidence intervals were calculated using the bootstrap method, implemented in a custom R script. However, the “boot” package was used to the check some of the confidence intervals obtained. Finally, multiple testing correction was performed using the p.adjust function available in base R, using the “BH” (Benjamini-Hochberg) parameter.

The comparison of estimated doping prevalences in Daegu and Moscow, performed using a Kolmogorov-Smirnoff and a Cramér von Mises test, used the standard R functions for the former, and the “goftest” package for the later.

References

R: A language and environment for statistical computing. R Foundation for Statistical Computing [2005. Vienna, Austria.

Robinson N, Saugy J, Schutz F, Faiss R, Baume N, Giraud S, Saugy M. 2019. Worldwide distribution of blood values in elite track and field athletes: Biomarkers of altered erythropoiesis. Drug Test Anal. Apr;11:567-577. Epub 2018/10/23.

Schütz F, Zollinger A. 2018. ABPS: An R Package for Calculating the Abnormal Blood Profile Score. Frontiers in physiology. 2018-November-21;9.
